# Designing studies for post-treatment Lyme disease and other infection-associated chronic illnesses

**DOI:** 10.1093/brain/awag016

**Published:** 2026-05-18

**Authors:** Paul M Arnaboldi, Jacqueline Becker, Avindra Nath, Patricia K Coyle, Andrew Handel, Timothy J Sellati, Maria Gomes-Solecki, Sandra Garcet, Marianne K Henderson, Piper Mullins, Elliot Cowan, W Richard McCombie, Anna-Marie Wellins, Mark Allegretta, Jonas Bergquist, Steven E Schutzer

**Affiliations:** Department of Pathology, Microbiology, and Immunology, New York Medical College, Valhalla, NY 10595, USA; Department of Medicine, Ichan School of Medicine, New York, NY 10029, USA; National Institute of Neurological Disorders and Stroke, National Institutes of Health, Bethesda, MD 20892, USA; Department of Neurology, Stony Brook University Health Sciences Center School of Medicine, Stony Brook, NY 11794, USA; Department of Pediatrics, Stony Brook University Health Sciences Center School of Medicine, Stony Brook, NY 11794, USA; DiaSorin, Inc., Stillwater, MN 55082, USA; Department of Microbiology, Immunology, and Biochemistry, University of Tennessee Health Science Center College of Medicine, Memphis, TN 38163, USA; Center for Clinical and Translational Science, Rockefeller University, New York, NY 10065, USA; National Cancer Institute, National Institutes of Health, Bethesda, MD 20892, USA; Pan-Smithsonian Cryo-Initiative, Smithsonian Institution, Washington, DC 20013, USA; Partners in Diagnostics, Rockville, MD 20850, USA; Cold Spring Harbor Laboratory, Cold Spring Harbor, NY 11724, USA; Stony Brook School of Nursing, Stony Brook University, Stony Brook, NY 11794, USA; National Multiple Sclerosis Society, New York, NY 10017, USA; Department of Chemistry—BMC, Analytical Chemistry and Neurochemistry, Uppsala University, Uppsala SE-751 24, Sweden; ME/CFS Collaborative Research Centre, Uppsala University, Uppsala SE-751 24, Sweden; Department of Medicine, Rutgers New Jersey Medical School, Newark, NJ 07103, USA

**Keywords:** post-treatment Lyme disease syndrome, long COVID, myalgic encephalomyelitis/chronic fatigue syndrome, multiple sclerosis, infection associated chronic illness

## Abstract

Infection-associated chronic illnesses (IACIs) encompass a spectrum of poorly understood syndromes often marked by significant neurologic and multisystem symptoms following an infectious event.

This review focuses on several diseases representative of the IACI spectrum. These are post-treatment Lyme disease syndrome (PTLDS), long COVID, myalgic encephalomyelitis/chronic fatigue syndrome (ME/CFS) and multiple sclerosis (MS). Their clinical and biological complexity, combined with a lack of clear diagnostic criteria and objective available laboratory biomarkers, makes them difficult to distinguish from conditions with overlapping features. This presents challenges for research studies, as well as diagnosis and clinical management. This diagnostic ambiguity, coupled with heterogeneous patient presentations, has led to challenges in research, including misclassification of study participants and inconsistent or irreproducible findings. Some PTLDS research exemplifies these issues, which also extend to other IACIs.

To advance the field, we highlight key methodological refinements and approaches for studying IACIs, including rigorous participant selection, standardized sample collection protocols, and the use of appropriate control groups, including those with microbiologic proof of the initial infection when known and technologically feasible. We also address broader influences on research quality, such as stigma, historical neglect, and the urgency to find treatments, which have contributed to the proliferation of poorly controlled studies and questionable practices. Drawing lessons from past challenges, we propose a path forward grounded in fit-for-purpose methodological rigour to improve scientific understanding and support evidence-based therapeutic development for IACIs.

## Teaser

Arnaboldi *et al.* outline key principles for studying infection-associated chronic illnesses, including post-treatment Lyme disease syndrome and long COVID. They emphasize the need for fit-for-purpose study design, rigorous participant selection, and appropriate controls to reduce misclassification and improve reproducibility.

## Introduction

Several distinct conditions appear to be triggered by infections that lead to chronic symptoms involving the nervous system. These conditions typically do not result from persistent infection and are unresponsive to antimicrobial treatments. Understanding one may shed light on others, especially if they share underlying neurologically mediated mechanisms. Many of the diseases are referred to as infection-associated chronic illnesses (IACIs). As representatives of the spectrum, we focus on post-treatment Lyme disease syndrome (PTLDS), long COVID, myalgic encephalomyelitis/chronic fatigue syndrome (ME/CFS) and multiple sclerosis (MS).

In the United States, Lyme disease is an infectious disease caused predominantly by the bacterium *Borrelia burgdorferi*. The cause for persistent symptoms remains a subject of ongoing debate due to conflicting findings and a wide range of clinical manifestations.^1-4^ While antibiotic therapy is effective for clearing the bacterium, some individuals experience persistent symptoms following treatment, with neurologic complications among the most prominent and disabling. This condition, referred to as post-treatment Lyme disease (PTLDS) (see later), differs from chronic Lyme disease (CLD), a term used by some to describe individuals with a vague array of symptoms, frequently lacking documentation of exposure to *B. burgdorferi.*^1-8^ The distinction between these terms is critical, as the lack of rigorous diagnostic criteria in past studies has hindered progress in understanding Lyme disease and its long-term sequelae.

Most prior studies have not required direct microbiologic proof of *B. burgdorferi* infection and have often omitted appropriate control groups. This has led to the possible inclusion of heterogeneous cohorts and the production of data that are difficult to interpret or replicate. Clarifying the full clinical picture of Lyme disease requires a cohort of study participants who are truly infected with *B. burgdorferi* and not co-mingled with another illness that closely mimics Lyme disease. It is both feasible and necessary to change our approach to truly understand this condition and find effective therapies for those who develop persistent sequelae. Otherwise, failing to correct past limitations will continue to stifle progress in the field. The methodological advances outlined in this paper address these past limitations.

In this review, we focus on and refer to PTLDS using criteria proposed and generally accepted by most academic and government agencies.^4,9-11^ Individuals with PTLDS had laboratory and clinically supported evidence of *B. burgdorferi* infection. They received appropriate early antibiotic therapy^9,12^ and continued to have or develop new symptoms within 6 months of infection that lasted for a period of at least 6 months, resulting in impaired or declining function. In PTLDS and other IACIs, several risk factors, including delays in antibiotic treatment, higher symptom severity in early disease, higher bacterial load, older age, comorbidities and female sex are found.^4,13^ In IACIs, the most common symptoms are neurologic-associated. These include cognitive impairment, fatigue and pain.^2-4,7,8,10,11,14^

The non-specific presentations of these conditions have historically contributed to controversy and scepticism within the medical community, often leaving individuals feeling dismissed or misunderstood. This has led some individuals to seek non-evidence-based and, at times, unsafe alternative therapies to alleviate their symptoms.^15^

The challenges posed by the non-specific nature of symptoms in PTLDS and other IACIs are further compounded by uncertainties surrounding their underlying causes. While PTLDS and long COVID have specific infectious triggers, the role of infection in conditions like MS and ME/CFS remains a subject of investigation (Table 1). In ME/CFS, there is no proven single microbial cause, nor is there any specific therapy for the disease. In MS, Epstein-Barr virus (EBV) infection is a necessary environmental factor.^16,17^ For this reason, MS and ME/CFS are often included in discussions of IACIs. Unlike PTLDS and ME/CFS, MS has objective physical examination and laboratory (such as CSF oligoclonal bands, kappa free light chain index^18^) and imaging findings and has been the subject of scientific investigation for nearly two centuries. MS, in contrast to other IACIs, is associated with objective diagnostic criteria that have enabled the design of well-controlled, rigorous and reproducible clinical studies, leading to the development of effective, US Food and Drug Administration (FDA)-approved disease-modifying therapies.^19-21^ The stakeholder community of individuals with MS, researchers, clinicians, insurance providers and MS societies has accepted these studies and disease-modifying therapies and promoted their use for patients. This disease could shed light on other presumptive IACIs, because it demonstrates that, even when the originating cause is unproven, properly conducted clinical and basic science investigations can result in FDA-approved treatments that improve the lives of individuals with MS.^22-26^

**Table 1:** Comparison of diseases with cognitive dysfunction and fatigue

Disease	Known initial infectious cause	FDA-approved therapy
Post-treatment Lyme disease syndrome	Yes, *Borrelia burgdorferi*	No
Myalgic encephalomyelitis/chronic fatigue syndrome	No	No
Multiple sclerosis	No (but prior Epstein-Barr virus infection required)	Yes
Long COVID	Yes, SARS-CoV-2	No

FDA = US Food and Drug Administration.

The authors of this article are researchers and physician-scientists representing diverse disciplines, including immunology, infectious disease, neurology, paediatrics, primary care, neuropsychology, biorepository curation and public health, each bringing complementary expertise to the study of IACIs. This paper was inspired by discussions held at a meeting at The Cold Spring Harbor Laboratory Banbury Center in late 2024. Our purpose is not to provide a detailed review of PTLDS or IACIs, as this is done elsewhere^4,11,13^ and commonalities are abundant, but rather to discuss important topics for constructing studies for these diseases. Neither the meeting nor this paper aims to establish a consensus on the study of IACIs. Rather, it offers guidance and best practices for the design and implementation of clinical trials in PTLDS and other IACIs characterized by neurologic symptoms of fatigue and cognitive impairment.

We emphasize the need for fit-for-purpose^27^ (intended use) criteria, which apply to both the methodology and reason behind a study. A treatment study does not have to eliminate all symptoms to be effective. The purpose of this article is to examine key investigative principles that can drive the development of effective, credible therapies, while avoiding the methodological pitfalls that have historically led to inconclusive or uninterpretable data.

There is an urgent need for effective treatments for individuals who are suffering, whether it be curative or disease-modifying. The strong desire for positive outcomes often leads to the premature adoption of drug therapies or other interventions that lack rigorous evaluation, which almost universally leads to temporary and limited benefits or outright failure. Flawed efforts leave patients discouraged and do not meaningfully contribute to health advancements.^15^

## Overviews of post-treatment Lyme disease syndrome and other IACIs

### Post-treatment Lyme disease syndrome

In the USA, Lyme disease was first recognized as a distinct entity in the 1970s, when a mother in Connecticut alerted the medical community to an unusual appearance of oligoarticular arthritis in contiguous communities near Lyme, CT.^28^ This eventually led to the discovery of its association with tick bites carrying the causative bacterium, *B. burgdorferi.*^29-31^ However, PTLDS was slow to gain acceptance, even as research on the objective neurologic manifestations in people with confirmed acute Lyme disease progressed.^32-35^

The exact number of PTLDS cases is unknown, and estimates are broad.^36-40^ Some literature suggests that 10%–20% of individuals who receive appropriate antibiotic therapy for acute Lyme disease are affected,^13,36,41^ while other sources suggest more conservative estimates of 5%–10%.^38-40,42^ Based on estimates from the Centers for Disease Control and Prevention (CDC) and others, this translates to thousands to tens of thousands of new cases annually. PTLDS is distinct from untreated or inadequately treated Lyme disease, in which individuals either do not receive antibiotics or fail to complete their prescribed regimen, resulting in possible continued infection.^43,44^ People with PTLDS are sometimes included in a group, more colloquially referred to as chronic Lyme disease,^4^ which includes individuals who may have fewer or no objective laboratory signs of *B. burgdorferi* infection or exposure. Unlike untreated Lyme disease, studies have consistently demonstrated that prolonged antibiotic therapy provides no sustained benefit for PTLDS,^4^ making the hypothesis that active infection is associated with PTLDS unlikely.

Individuals with PTLDS experience persistent neurologic-associated symptoms, most commonly cognitive impairment, profound fatigue and diffuse pain. These symptoms typically emerge within 6 months of a *B. burgdorferi* infection and can persist for months to years, significantly impacting daily function.^4,9-11^ While *B. burgdorferi* infection is considered the initiating event, the underlying mechanisms driving persistent symptoms are unknown. Immune dysfunction is suspected to play a central role, yet no definitive mediator has been established.^10,11^ Current studies of PTLDS suggest it may arise from an interplay of factors, including persistent immune activation, lingering bacterial antigens, or other yet-to-be-defined mechanisms that prolong or reignite an inflammatory response.^45-50^ However, in most studies, very few individuals with definitive microbiologic proof of *B. burgdorferi* infection are included. Rather, clinical evidence (a skin lesion or serology) suggestive of Lyme disease has most often been used as a study enrolment criterion (see the ‘Challenges of studying post-treatment Lyme disease syndrome and other IACIs’ section for limitations). Furthermore, some studies include patients without objective evidence of Lyme disease. Thus, it is not clear that every patient included in studies has or had Lyme disease, which results in confounded data. No effective treatment options currently exist for PTLDS. This may be due to the incomplete understanding of the condition’s pathophysiology. A deeper understanding of these immunopathogenic pathways can help the development of targeted diagnostic approaches and therapies.

### Multiple sclerosis

MS is recognized as a complex, immune-mediated neurological disorder characterized by inflammation, demyelination and neurodegeneration. Cognitive dysfunction and fatigue are common features of the disease, a connection first noted by Charcot,^51^ who described these as ‘enfeeblement of memory’ and ‘concepts formed slowly’.

Current understanding suggests that MS is initiated by a triggering infection, although it typically manifests years later as a lifelong immune-mediated CNS disorder. Chronic symptoms of fatigue, cognitive disturbance, pain and mental health conditions are commonly observed. MS is estimated to affect about one million Americans and close to three million individuals worldwide.^52,53^ It primarily emerges in young people, with onset at or following puberty, and approximately 90% of individuals present between the ages of 15 and 50 years of age. There is a modest but increasing female predominance, now showing a 3:1 female-to-male ratio.^20,54^

MS is a highly variable disorder with highly variable presentations. It is believed that individuals with MS have a genetic predisposition, i.e. too many risk/susceptibility genes and/or too few disease protection genes, with recognized low, medium and high-risk geographic latitude zones. Some cases remain clinically silent; autopsy studies indicate up to 25% of those with pathologic MS have no clinical history.^55^ Radiologically isolated syndrome (RIS) refers to asymptomatic individuals whose MRI scans, conducted for unrelated reasons (such as trauma or headache), reveal lesions highly suggestive of MS.^56,57^ In a 10-year follow-up study, more than 50% of RIS subjects had clinical MS onset of relapsing or primary progressive disease.^56^ The new expected revised MS McDonald diagnostic criteria^58^ will allow RIS to be diagnosed as MS.

The MS disease course consists of two major components: relapsing and progressive.^59,60^ Relapsing MS is characterized by focal inflammation, where immune cells cross the blood–brain barrier, triggering episodic attacks and contrast-enhancing macroscopic lesions on MRI. Adaptive immunity, as a contributory factor, is maximal at younger ages or in earlier disease stages but fades over time. Progressive MS, in contrast, is characterized by neurodegeneration (injury to synapses, axons and neurons), with smouldering CNS innate inflammation. This includes activated toxic microglia and astrocytes. Both adaptive and innate CNS inflammatory processes are believed to be present in most, if not all, individuals with MS. Manifestation of progressive MS is most common at mid-life (ages 45–55 years), presumably when sufficient CNS reserve has been lost, resulting in a clinical impact of gradual worsening, which manifests most prominently in altered gait and leg strength weakening. MS is now recognized to have a prodromal period of 5 to as long as 15 years, during which there is significantly greater than normal healthcare use compared to the control group not destined to develop MS.^61^

The precise aetiology of MS remains unknown. While genetic susceptibility contributes to disease risk, MS is clearly not an inherited disease. Environmental factors play a critical role, and mounting evidence suggests an infectious trigger is required. This makes MS an interesting example of a major neurologic disease that requires an infectious trigger, which subsequently leads to immune-mediated disease. The most well-described association is infection by EBV, which confers a 32-fold increased risk of developing MS.^16^ It appears that adult-onset MS requires prior EBV infection. Besides EBV, human endogenous retrovirus and human herpes virus type A have been implicated in the development of the disease.^62^ Individuals with MS have a generally higher rate of infection than non-MS individuals.^63^ Infection, even trivial respiratory tract pathogens, can trigger MS relapses and pseudo-relapses (temporary worsening of symptoms without real myelin inflammation or damage, due to other influences).

Despite its complex pathogenesis, MS has become a model for the successful development of FDA-approved disease-modifying therapies. Multiple FDA-approved treatments, primarily immunosuppressive, have demonstrated efficacy in managing relapsing forms of the disease, although they are noted to increase risk of infection.^19-26^ MS produces highly suggestive clinical and laboratory abnormalities that may provide valuable insights into other immune-mediated conditions triggered by infection.

### Long COVID

Long COVID, a condition only recently recognized as a distinct clinical entity, emerged during the COVID-19 pandemic. As an IACI, it is marked by persistent symptoms following infection with SARS-CoV-2 but otherwise lacks a clear definition. There have been several attempts to define long COVID^64^; however, with a multitude of subjective symptoms and the absence of defined laboratory diagnostic biomarkers (see later), a uniform diagnosis has been challenging. For research purposes, there is a consensus that the optimal definition should include laboratory confirmation of SARS-CoV-2 infection, followed by persistent or new symptoms lasting longer than 3 months that cannot be explained by other medical diagnoses or end-organ damage from acute COVID-19.

It is estimated that nearly 20 million people in the USA have long COVID, with about 25% of these individuals experiencing significant limitations to daily living.^65^ By 2022, the economic costs of long COVID in the USA alone were estimated to be in the trillions of dollars.^66^ Despite a lack of established biomarkers or disease-modifying therapies, substantial progress has been made in understanding the condition within just a few years. By identifying various distinct phenotypes and investigating the multiple mechanisms underlying its pathophysiology, researchers are beginning to grasp the heterogeneity of the illness. Emerging evidence suggests that the clinical phenotypes and underlying pathophysiological mechanisms of long COVID may be relevant to other post-infection syndromes or IACIs. Hence, treatments developed for long COVID could be useful in treating many of these other conditions.

Individuals with long COVID can largely be grouped into four different but overlapping phenotypes.^67^

- Those with cognitive complaints, often referred to as brain fog. These may be associated with sleep disturbances and mood abnormalities.
- Exercise intolerance. This may be associated with post-exertional malaise.
- Dysautonomia. In some individuals, this may result in postural orthostatic tachycardia syndrome (POTS).
- Pain syndromes, including fibromyalgia.

Recently, additional phenotypes have been identified, underscoring the complexity of the disease and the challenges in its study.^68-70^ While it is likely that varying clinical phenotypes have distinct pathophysiological mechanisms, this remains to be fully determined. In the absence of disease-modifying therapies, treatment of long COVID is limited to symptom management.

Multiple mechanisms are under investigation in long COVID. These include :

- Persistent viral reservoirs. Active viral replication or isolation from organs at autopsy in individuals who have recovered from the acute phase of the illness has been demonstrated only rarely. In one study, SARS-CoV-2 was isolated and propagated from brain tissue obtained at autopsy from a single individual.^71^ This virus was sequenced and shown to have distinct mutations. However, to date, there has been no evidence for viral encephalitis with SARS-CoV-2. Virus cannot be isolated or demonstrated by PCR in the CSF of acutely infected patients with neurological manifestations. There is no evidence of pleocytosis in the CSF or of T-cell infiltration in the brain at autopsy, which would be expected if the brain were infected with a virus. Furthermore, to date, there has been no evidence of SARS-CoV-2 transmission via blood transfusion or organ donation. Prior infection with SARS-CoV-2 is not a contraindication to donating blood or organs. A clinical trial with Paxlovid in individuals with long COVID did not show any benefit, although treatment with Paxlovid in the acute phase of the infection can decrease the probability of developing long COVID.^72^ While the final word on Paxlovid and long COVID is yet to be written,^73^ these observations are reminiscent of other infection-associated chronic conditions where prolonged treatment with antimicrobial agents has no significant effect. It should be noted that persistence of SARS-CoV-2 following acute infection is more common in people with long COVID than in those who recover from acute COVID.^74-82^
- Persistent viral antigen. Several studies show that remnants of the virus, i.e viral proteins and RNA, can be detected in rectal mucosa,^83^ tongue papilla^84^ and other organs^85^ for months and even years after the acute infection. It is possible that the inability to completely eradicate these viral products may result in immune exhaustion and innate immune activation. This may drive the systemic symptoms and lead to organ-specific manifestations. However, the cause and effect have yet to be determined. It is not unusual for RNA viruses to persist in this manner, which has been studied extensively in the context of Sindbis virus (reviewed by Griffin^86^), Ebola^87,88^ and other viruses.^89^ A similar mechanism has been proposed for PTLDS.
- Reactivation of herpes viruses. A few studies have reported EBV reactivation in individuals with long COVID, but the significance of this reactivation remains to be studied.^90,91^
- Persistent immune activation. One prevailing hypothesis is that following an acute infection, there is persistent immune activation, despite viral clearance. The hypothesis is that the immune system, for some reason, does not shut down, even though the virus has been cleared. There is ample evidence in support of persistent innate immune activation in individuals with long COVID.^92,93^

The use of complex cohorts, with perhaps different aetiologies following SARS-CoV-2 infection, makes comparing results across studies and identifying specific disease mechanisms and biomarkers a significant challenge.

### Myalgic encephalomyelitis/chronic fatigue syndrome

Historically, ME/CFS has been a highly controversial diagnosis, only gradually gaining broader acceptance within the medical community in recent years. With the emergence of long COVID, which has a clearly defined viral aetiology, the connection between chronic post-infectious diseases is receiving renewed attention. ME/CFS is a common clinical presentation in long COVID, where an estimated 4.5% or more of individuals who become infected with SARS-CoV-2 subsequently develop ME/CFS.^94-98^ ME/CFS also shares key neurologic features with PTLDS, particularly in its hallmark symptoms of profound fatigue, post-exertional malaise and cognitive impairment.

ME/CFS is now strongly suspected to be an IACI, and research has identified possible underlying mechanisms that align with other IACIs. Its onset has also been linked to immune dysfunction, environmental factors and traumatic events. However, in the absence of a single definitive inciting infectious agent, the disorder remains without a proven single origin or effective treatment. There may be multiple inciting or propagating infectious agents, including SARS-CoV-2.^99,100^ Diagnosis is typically delayed (sometimes years after the initial onset of symptoms), at which point evidence of cause(s) and early natural history has been lost. The diagnostic challenge mirrors those emerging in long COVID, where overlapping risk factors and related conditions are increasingly coming into focus.

Research increasingly suggests that ME/CFS may arise from a convergence of immune, neurologic, metabolic and genetic influences following an initial insult. Proposed mechanisms include immune and metabolic dysregulation, neuroendocrine abnormalities, nervous system and circulatory system dysfunction, cardiovascular defects and disturbances of the gut microbiome and intestinal barrier function.^101-105^ Possible aetiologies include immune system dysfunction that could be due to viral persistence, viral reactivation, B cell-, T cell- and/or NK cell-dysfunction and strong evidence for T cell exhaustion.^101,102^ In addition, CNS dysfunction resulting in cognitive impairment or ‘brain fog’ may be due to neuroinflammation and blood–brain barrier dysfunction. Autonomic nervous system and circulatory system dysfunction result in cardiovascular manifestations (heart rate variability), endothelial dysfunction and micro-clots. There is growing evidence of genetic risk factors and/or genetic susceptibility.^104^

### Other IACIs

Other diseases with objective diagnostic criteria can be considered IACIs, such as *Helicobacter pylori* infection, responsible for gastric and intestinal ulcers.^106,107^ This has been associated with cognitive impairment in some studies, whether due to brain-gut-microbiome dysbiosis, poor vitamin absorption or another mechanism. It is also an example of non-gastrointestinal symptoms that are likely due to a reaction (inflammatory or otherwise) to bacterial components rather than direct bacterial invasion.

## Challenges of studying post-treatment Lyme disease syndrome and other IACIs

Clinical trials and studies play an essential role in delineating disease pathophysiology and underlying mechanisms, as well as in assessing novel therapies and innovative methods for disease detection, diagnosis and risk reduction. These studies facilitate the discovery and evaluation of diagnostic and prognostic biomarkers, the safety profiles of specific pharmaceuticals, optimal dosages and treatment efficacy, and the reasons for variability in responses. Well-designed clinical trials enable healthcare practitioners and patients to make informed decisions about balancing the adverse effects of new interventions with anticipated benefits.

The success of a clinical trial, or any clinical study, depends on the study design. For IACIs, study design can be particularly challenging (Table 2). The choice of appropriate (fit-for-purpose) inclusion and exclusion criteria is critical. Cases need to be selected according to precise criteria, particularly for IACIs, and reliable testing for infectious agents. Within a group of specific individuals under investigation, heterogeneity should be kept to a minimum. Too much variation within cohorts can influence a trial’s outcome by possibly obscuring treatment effects in subgroups that may react differently to the intervention. Differences in disease characteristics (e.g. severity, stage and presence of co-morbidities) and demographic, genetic and lifestyle factors are common sources of heterogeneity and must be taken into consideration when analysing results within and between study groups.^108,109^ Subgroups, such as those with differences in underlying biological abnormalities or responses to treatment or prognoses, identified within the larger case group, should be analysed separately.

**Table 2:** Commonly overlooked methodological issues in IACI research

Methodological issue	Description	Recommendations
Study participant enrolment considerations	Studies often include highly heterogeneous participants with varying clinical presentations and insufficient stratification based on disease mechanisms. This can complicate biomarker discovery and obscure treatment effects.	Minimize cohort heterogeneity. Careful selection of inclusion/exclusion criteria. Stratify based on disease mechanisms. Standardize enrolment protocols. Separate highly heterogeneous and more homogenous groups into different cohorts
Unique challenges in measuring cognitive dysfunction	Studies vary with respect to the measures used, often do not account for confounding factors, fail to incorporate performance validity measures, and include diverse populations.	Identify common data elements for cognitive assessments. Incorporate performance validity measures. Account for confounding factors. Ensure normative data. and that study designs reflect susceptible populations.
Inclusion of biologically relevant control groups	Studies often lack infection-exposed, asymptomatic controls, making it difficult to isolate disease-specific responses.	Include both healthy controls and infection-exposed, asymptomatic controls to improve the validity and interpretability of results.
Variable of time since infection	Time elapsed since infection is frequently not accounted for, which can lead to misinterpretation of biomarker persistence and disease specificity.	Match cases and controls by time since infection or use longitudinal study designs to track changes over time.
Sample collection intricacies	Variability in sample collection, timing, and handling can impact biomarker measurements, particularly for biomarkers with circadian rhythms.	Standardize collection protocols, use multiple time points for biomarkers with known fluctuations, and ensure methodological consistency, especially in multicentre studies.

IACI = infection-associated chronic illness.

Although of suspected distinct aetiology, PTLDS, long COVID, ME/CFS and MS all share primary symptoms that are neurologically associated, non-specific and/or subjective in nature. However, MS has more objective physical exam, laboratory and imaging findings than the other diseases. Fatigue, cognitive impairment (or the colloquially termed brain fog) and pain are common symptoms of a wide range of conditions, syndromes and diseases. Relying solely on these features for diagnosis results in an overwhelmingly broad differential, making it impossible to distinguish potential underlying causes and creating significant challenges in defining inclusion/exclusion criteria for studies of IACIs. This takes on special relevance in the age of systems biology and -omics studies, which examine global changes in the transcriptome, proteome, metabolome and microbiome, amongst others, over the course of a disease. These technologies produce massive datasets that must be parsed bioinformatically to identify common biomarkers and mechanisms underlying specific diseases. If a study enrols a co-mingled heterogeneous population containing individuals with different possible disease aetiologies that produce similar symptoms, the resultant data would likely be uninterpretable. This problem is exacerbated by additional factors such as: site-to-site variability in study participant enrolment when strict empirical evidence of disease is not available or used; inappropriate or insufficient controls; lack of appropriate statistical analysis; and failure to consider variances in sample collection, including differences in sample handling and storage procedures. These factors need to be addressed in the planning stages and should be done in consultation with a statistician from the inception stage. It is recognized that MS is distinct, with longstanding diagnostic criteria that are periodically revised.

With PTLDS, methodological challenges are particularly evident in studies aimed at identifying biomarkers and elucidating pathophysiological mechanisms. Establishing homogeneous cohorts for PTLDS research presents significant, but not insurmountable, challenges. Diagnosing early Lyme disease itself can be difficult, as definitive microbiologic confirmation of *B. burgdorferi* infection is rarely obtained in clinical settings. Instead, diagnosis is largely clinical, based on the presence of an erythema migrans (EM) lesion, accompanying symptoms, a history of tick exposure (with or without a known tick bite) and supportive serologic testing.^12^ However, reliance on visual inspection of EM is not pathognomonic, because other skin lesions can mimic EM.^110,111^ Without validated microbiological proof of infection (culture, DNA or detection of distinct *B. burgdorferi* antigenic sequences), the non-specific symptoms present in early Lyme disease and PTLDS can easily lead to mischaracterization and erroneous inclusion of individuals with other conditions into a study. When combined with the use of insufficient comparator groups (controls, see later), it has been challenging to compare and integrate data from different studies, which have enrolled and characterized individuals using vastly different criteria.

As a more recent clinical entity, studies on pathophysiological mechanisms in long COVID have similar challenges. These studies largely focus on viral and immune abnormalities but typically include a mix of individuals from the various phenotypes described earlier. Unsurprisingly, multiple mechanisms have been implicated. Recent publications on ME/CFS and long COVID discuss these and other challenges.^81,99,100,112-117^

### Challenge of identifying study participants for research

To advance the development of improved diagnostic tests and effective treatments for PTLDS, it is crucial to distinguish clearly between individuals with confirmed Lyme disease and those whose clinical presentation resembles PTLDS. Retrospective analyses often struggle to achieve this distinction. Carefully designing prospective studies that include a group with definitive proof of *B. burgdorferi* infection as an enrolment criterion is essential to mitigating the limitations of past research. Although *B. burgdorferi* may only be present transiently in the blood and in very low concentrations, it can frequently be found in a skin biopsy of an EM lesion.^118^ While skin biopsies are not used for routine clinical care, they are minimally invasive procedures that are imperative to defining a population for a research study. There are look-a-like lesions that are indistinguishable from EM, which could lead to the inclusion of a percentage of study participants with other diseases in the analysis.^110,111^ Metagenomics^119,120^ and antigen detection methods^121-123^ are new and promising techniques for the direct detection of *B. burgdorferi*, but these are still under development. In the absence of direct measurements, clinical suspicion of neurologic Lyme disease involving the PNS or CNS should be confirmed by at least one laboratory test.^124^ Clinical practice guidelines recommend detection of *B. burgdorferi*-specific antibody in serum and/or CSF,^12,125^ i.e. a CSF index, often through the CDC-recommended two-tier algorithm for serologic tests.^126,127^ However, as serologic tests do not directly detect the pathogen, individuals with seropositivity may have had a distant rather than recent infection, warranting their independent evaluation in research studies. Other indirect diagnostic approaches, such as gamma interferon release,^128,129^ CXCL13 detection,^130^ metabolomics^131,132^ and CSF proteomics,^35^ offer potential insights but are not FDA-approved or fully validated for specificity.^133^ In addition, ancillary testing, such as brain MRI, can help to rule out other causes of neurologic symptoms, including MS.

In the case of long COVID, demonstrating SARS-CoV-2 as an antecedent or measuring post-exertional fatigue can provide valuable context. Long COVID is somewhat easier to verify than PTLDS due to the availability of direct detection assays. However, the various phenotypes observed raise questions of whether different forms of long COVID have different aetiologies. Careful characterization or analysis of study participants is needed in all cases of IACIs to limit variables that confound data interpretation.

### Unique challenges in measuring cognitive dysfunction

The neurologic sequelae across IACIs underscore the profound impact of viral and/or bacterial infections on the CNS.^134^ Cognitive ability, a multidimensional construct encompassing domains such as memory, attention, executive functioning and processing speed, among others, serves as a critical marker of neuronal function and integrity. Evaluating cognition in the context of IACIs such as long COVID, PTLDS or ME/CFS is essential to understanding the breadth of CNS involvement, as well as for tracking persistence or recovery over time and tailoring interventions to improve quality of life.^135,136^ However, research into the assessment of cognitive dysfunction associated with IACIs continues to face considerable challenges.^135,136^

IACIs often manifest with highly variable and non-specific symptoms.^136^ Cognitive dysfunction includes self-reported or objective (formally measured) cognitive deficits, most often causing some degree of functional impairment.^137^ While most studies have demonstrated predominant impairments in aspects of executive functioning, these impairments vary in severity and presentation and may span different cognitive domains.^138,139^ Cognitive symptoms are believed to arise from widespread CNS inflammation, immune dysregulation or direct effects on neuronal and glial cells. A comprehensive understanding of CNS involvement necessitates cognitive assessments that are both sensitive to individual nuances and capable of capturing the variability in symptom presentation. However, achieving this level of precision is often challenging within the constraints of research studies.

An additional challenge is that studies vary widely in the measurement methods they employ. The lack of standardization in cognitive assessment protocols has resulted in inconsistency in the estimated prevalence and characterization of cognitive dysfunction in IACIs. Measurement tools include brief cognitive screeners (designed to detect dementia but insensitive to detecting the often subtle impairments associated with IACIs),^140^ online surveys (which frequently lack adequate reliability and validity),^141^ self-report questionnaires (which may be prone to biases and are often discordant with objectively measured deficits)^142^ and finally, comprehensive and validated neuropsychological measures (the gold standard for assessing cognition, yet infrequently utilized).^138,139^ There is no question that validated neuropsychological measures should be utilized over cognitive screeners that lack the sensitivity needed to detect impairment in IACIs; however, even in studies utilizing validated neuropsychological measures, there is considerable variability in the selection of normative data, which may not entirely be generalizable to individuals with IACIs or those of different cultural backgrounds. Studies also vary in their consideration of performance validity tests (PVTs), which should be standard in every cognitive assessment.^143^ Importantly, cognitive measures may occasionally fail to capture the often subtle deficits in IACIs beyond a clinical threshold, sometimes due to the episodic nature of symptoms.^144^ Thus, when possible, cognitive testing should typically include full-scale neuropsychiatric test batteries, rather than less precise screening tests.

Cohort selection also remains a significant challenge in research on cognitive dysfunction related to IACIs. Many epidemiological studies are affected by selection biases, often focusing on individuals actively seeking care for cognitive complaints.^145^ Another critical issue is establishing appropriate comparator or control groups. Differentiating the cognitive effects of IACIs from those of healthy individuals or those with similar symptoms stemming from different causes (e.g. post-concussion syndrome) demands careful selection of control or comparison groups. Additionally, the interpretation of findings must consider confounding factors, such as pre-existing cognitive vulnerabilities, cognitive effects of medications used to manage IACI symptoms, and other common IACI conditions or symptoms, such as fatigue, pain, depression, or anxiety, which can independently impact cognitive function.^135^

In summary, the heterogeneous presentation of IACIs necessitates nuanced approaches to the assessment and interpretation of cognitive functioning. Identifying common data elements for cognitive assessments by standardizing neuropsychological testing protocols, incorporating performance validity measures and accounting for confounding factors are necessary steps to improving our understanding of the cognitive effects of IACIs. Future research must also prioritize ensuring that normative data and study designs reflect susceptible populations, addressing differences in access to care and capturing the lived experiences of individuals with IACIs. Ultimately, a nuanced and multidisciplinary approach to cognitive dysfunction in IACIs will allow us to identify factors that predict recovery over time and provide the foundation for more effective interventions to improve quality of life for affected individuals.

## Other considerations

The importance of a carefully designed study cannot be overstated. Many individuals suffering from long-term symptoms associated with IACIs are desperate for relief. In CLD and PTLDS, many people are eager to participate in studies to evaluate alternatives to antibiotic treatment. Many purportedly effective treatments for CLD depart from mainstream medical practices and broadly fall into several categories, including: (i) prolonged and unorthodox antibiotic regimens; (ii) oxygen and reactive oxygen species; (iii) energy and radiation; (iv) heavy metals and chelation; (v) nutrients; and (vi) biological, pharmacological, stem cell and herbal therapies.^15,146^ Some alternative treatments pose significant health risks and financial burdens, with no rigorous clinical validation of effectiveness or evidence of benefit, beyond testimonials and the promotional materials of health practitioners treating individuals with CLD.^15^ It is common to see extrapolated findings from *in vitro* or animal studies of clinical treatment, often in the absence of rigorous human trials. One example is the use of ozone treatments. Some *in vitro* and animal studies, along with case reports, suggest that oxygen-based treatments (e.g. hyperbaric oxygen, ozone and hydrogen peroxide) may improve the well-being and quality of life of individuals with CLD.^147-150^ Proponents have suggested that reactive oxygen species, such as ozone, can be delivered in various ways: via intravenous ozonated solutions, drinking ozonated water, ozonating and reinfusing blood, using ozonated oils, and administering gaseous ozone via rectal or vaginal insufflation.^147-150^ However, there is a critical lack of well-designed, large-scale clinical trials to substantiate the clinical benefits of oxygen or reactive oxygen species. Paramount would be definitive criteria that would establish a uniform study cohort associated with *B. burgdorferi* infection. Without this, potential benefits could be obscured by the heterogeneity of individuals with similar symptoms of different aetiologies. Such a treatment might appear effective in some individuals and not others simply because their symptoms have a different underlying cause. In the absence of robust evidence, recommending these treatments is challenging, particularly given the potential for significant negative health consequences.^15,146,151,152^

There are currently no disease-modifying therapies available for long COVID or ME/CFS. Instead, management relies on a wide array of symptomatic treatments. In a desperate search for relief, many individuals will turn to unproven and often questionable treatments, risking harm rather than benefit. However, such cases are rarely documented in the medical literature. Few placebo-controlled trials have been conducted. Among them, studies investigating intravenous mesenchymal stem cells^153^ and an antibody targeting the human endogenous retrovirus-W envelope protein, activated in monocytes of individuals with acute COVID, failed to meet their end points. A key limitation of these trials was the lack of study participant stratification based on pathogenic mechanisms; instead, participants were included based solely on broad eligibility criteria, obscuring potential benefits if there were any.

To advance treatment, future clinical trials should enrol individuals according to the specific pathogenic mechanisms targeted by the intervention. For example, if an antiviral drug is to be tested, only individuals with evidence of viral activation should be enrolled. There is vast potential to examine immunotherapies in this population, including toll-like receptor agonists, anti-B cell therapies, checkpoint inhibitors, anti-immune senescence therapies, and drugs that block innate immune activation. A deeper understanding of distinct clinical phenotypes and their underlying mechanisms will be critical to designing effective, targeted therapies.

### Use of biologically relevant control groups

As research into IACIs advances, the overarching goal is to identify biomarkers, mechanisms and clinical characteristics that define persistent symptoms following infections. However, methodological limitations, particularly in the selection of appropriate control groups, continue to challenge the reliability and interpretability of findings. Control groups should include not just healthy individuals but also: (i) people who have experienced the same infection but not developed a chronic illness (symptom resolution); and (ii) people with other diseases that can cause the same symptoms (symptomatic controls). Establishing biologically relevant control groups requires careful consideration of factors such as the timing of assessments relative to the initial infection, methodological consistency, and biological variables in sample collection. This section discusses each of these, drawing examples from recent studies to illustrate their impact and offering recommendations for improving future research.

The identification of biomarkers that aid diagnosis, disease monitoring or mechanistic understanding is essential. We refer to biomarkers as outlined in the FDA-NIH ‘BEST’ (biomarkers, endpoints, and other tools) resource.^154^ Biomarkers document objective pathology and can have different purposes, such as being diagnostic, prognostic or indicating response to therapy. Biomarkers can be the traditional laboratory-type or in some cases be of the observation-phenomena type. Caution is imperative with the use of a sole observation, such as EM for Lyme disease, where many mimickers exist. Although many studies include healthy individuals as controls, these groups often lack a history of infection similar to those with IACIs. As a result, they fail to adequately account for the biological changes triggered by infection itself to provide a meaningful comparison and help isolate disease-specific responses. For example, a study by Kim *et al*.^155^ examined the presence of autoantibodies against peptidylarginine deiminase 2 (PAD2) in individuals with PTLDS.^155^ However, the study did not include a group of individuals who had recovered from Lyme disease without persistent symptoms. Without this infection-exposed but asymptomatic group, it is difficult to determine whether the observed anti-PAD2 antibody levels are unique to PTLDS or are simply part of an immune response to *B. burgdorferi* infection.^155^ Similarly, Clarke *et al*.^156^ conducted a study to identify gene expression patterns associated with PTLDS using RNA sequencing data. The authors claimed to have identified an RNA signature that would predict PTLDS. However, the study design lacked a control group of individuals who had fully recovered from Lyme disease without persistent symptoms. The absence of this control group weakens the study’s conclusions, as any observed differences might not be due to PTLDS specifically, but rather to Lyme disease as a whole.^156^ In another study of individuals with PTLDS, it was suggested they exhibited a distinct pattern of gut microbiome dysbiosis. However, the comparator groups were healthy and intensive-care unit controls, leaving unanswered the question of whether the observed dysbiosis was unique to individuals with PTLDS or might also be found in those with early, acute disease.^157^

To improve the validity and interpretability of IACI research, future studies should not be restricted to healthy controls but have infection-exposed, recovered and asymptomatic controls, as well as mimicking disease controls. This approach would enable a more precise distinction between the effects of infection exposure and the IACI, ultimately improving the robustness of study findings.

### The time since infection variable

Another key consideration in IACI research is the time elapsed since the initial infection or treatment. The immune response and levels of various biomarkers can change significantly over time, which can confound findings if time since infection is not carefully controlled. Without appropriate time-matching, observed differences between groups may reflect natural recovery processes rather than disease-specific features.

An illustrative example of this limitation comes from a study by Swank *et al*.,^158^ which investigated the persistence of the SARS-CoV-2 spike protein as a potential biomarker for long COVID.^158^ Finding greater levels of persisting SARS-CoV-2 spike protein in circulation in individuals with long COVID, the authors conclude that the ‘presence of circulating spike supports the hypothesis that a reservoir of active virus persists in the body’. However, the study did not adequately control for time since infection. The samples included individuals at various stages of recovery from COVID-19, with no clear documentation of how long it had been since each participant’s initial infection. The lack of this crucial detail complicates the interpretation of the data, as the immune response and degree of protein clearance can differ based on how much time has passed since infection. Without matching cases and controls for time since infection, it is challenging to determine whether the spike protein is specific to long COVID or simply a residual marker that naturally declines over time after COVID-19 recovery.^158^

The temporal dynamics of biomarkers are especially important in IACI research, as the recovery process often involves gradual changes in immune function and biomarker levels. Matching cases and controls by time since exposure would help differentiate disease markers from those associated with normal post-infection recovery. Time since the acute infection (when available) should be included as a variable in any multivariable analysis of the data. In some cases, longitudinal study designs that follow participants over time are preferable, as they can capture individual variations in recovery trajectories and provide a more comprehensive understanding of biomarker changes over time. Of course, serial measurement of the same individual over time facilitates more robust statistical analysis of study results.

### Sample collection intricacies

Consistency in sample collection, transportation, handling and storage is crucial for accurate biomarker measurement in IACI research. Variations in collection methods, timing and processing can significantly impact biomarker data, potentially leading to misleading conclusions (presented in more detail later). This issue is even more relevant for biomarkers affected by biological rhythms, such as circadian or diurnal cycles. Failing to account for these natural variations can undermine data reliability^157^ and interpretation.

A recent study assessed cortisol levels in individuals with long COVID and compared them with levels in individuals who had recovered from COVID-19 without persistent symptoms.^91^ The study noted that cortisol levels were dramatically lower in individuals with long COVID, suggesting potential HPA (hypothalamic-pituitary-adrenal) axis dysfunction. However, the study did not adequately account for the natural fluctuations in cortisol levels throughout the day. Cortisol levels in the body change significantly during the day, following a distinct circadian rhythm. Cortisol typically rises rapidly in the first half hour after awakening and then declines sharply in the next few hours.^159^ In addition to this daily pattern, cortisol also displays ultradian rhythms, characterized by a series of smaller peaks and dips that occur at varying intervals and intensities throughout the day.^160^ Consequently, reliable assessment requires multiple samples collected at different times, rather than relying on a single measurement, to capture both the diurnal and ultradian variations in cortisol. Measuring cortisol at a single time point and without accounting for time since awakening, as was done in the study,^91^ fails to capture this diurnal pattern and leads to unreliable data. This challenge is further complicated by the potential link between long COVID and sleep disturbances,^161^ such as earlier-than-average wake times. These disruptions can shift the normal cortisol diurnal pattern, creating the impression of consistently lower cortisol levels throughout the day.

For IACI studies involving biomarkers with known diurnal or circadian patterns, researchers should use standardized collection times and multiple sampling points across the day to capture natural fluctuations. Time of day should also be included. In addition to controlling for biological fluctuations, consistency in other facets of collection methodology across all study participants is critical. Standardizing these protocols is particularly important in multi-centre studies, where variations in laboratory procedures are more likely. Further considerations for sample collection, transport and storage are considered later.

Understanding the pathogenesis of IACIs requires methodologically rigorous studies to yield reliable and interpretable results. Methodological improvements, such as using appropriate control groups, matching participants by time since infection and ensuring consistency in sample collection, are essential for enhancing the quality of research findings. Adopting these methodological practices can help to advance the field of IACI research, likely enabling the development of diagnostic tools and therapeutic interventions. Rigorous and consistent study designs will ultimately support the accurate identification of biomarkers, enhancing our understanding of IACIs, revealing potential therapeutic targets and improving outcomes for those impacted by these illnesses.

Rigorous and consistent study designs will ultimately support the accurate identification of biomarkers, enhancing our understanding of IACIs and improving outcomes. Often key methodological issues are overlooked or not accounted for.

### Studying post-treatment Lyme disease syndrome and other post-infectious syndromes in children

Paediatric clinical care often relies on results from adult studies due to a relative paucity of focused paediatric clinical trials. This is also true of many post-infectious syndromes, which have historically been understudied in young children and adolescents. Research on Lyme disease and its post-infectious complications is of particular importance to children, as the incidence of acute infections peaks at 5–9 years old.^162^ Clinical manifestations of Lyme disease also differ by age at the time of infection, as children are more likely to experience Lyme arthritis or facial nerve palsy but less likely to experience symptomatic carditis.^163-165^ Unlike adults, and despite the high incidence of paediatric Lyme disease infections, PTLDS is thought to be very uncommon in children.^166-168^ Yet our understanding of paediatric PTLDS is substantially limited, as few well-designed studies have investigated paediatric incidence and clinical phenotype. Similarly, childhood ME/CFS has only been recognized in recent decades despite paediatric prevalence estimates of 0.4%–2.4%.^169^ Paediatric long COVID has been more closely studied than other post-infectious syndromes, but to a lesser degree than in adults.^170^

Children differ from adults in a myriad of ways. These include rapidly changing metabolism, physiology and neurodevelopmental stages as children age, distinct disease manifestations, and the reliance on adults to meet their basic needs. Clinical research of acute illnesses and post-infectious syndromes in children, therefore, requires careful consideration of these distinctions, which should guide study design and implementation strategies.^171,172^ The conduct of successful clinical trials of post-infectious syndromes that produce meaningful findings must therefore address paediatric-specific research gaps, utilize child-friendly research tools, and be designed with the needs of child participants and their families in mind. With thoughtful planning and involvement of child and family advocates, large-scale research studies can be designed to address these concerns and avoid potential study failures (Table 3).

**Table 3:** Challenges and solutions for paediatric-specific challenges in studying post-infectious syndromes

Challenge	Potential solution
Broad range of neurodevelopmental stages in the paediatric population.	Consider the needs of each age group independently, adjusting assessments and interventions to be relevant for that population. Long-term follow-up studies must account for neurodevelopmental changes over time.
Fear of blood draws	Limit the number and volume of blood draws to only those necessary. Stagger venipuncture timing between study participants. Utilize paediatric-specific tools for successful blood draws.
Complicated school and family schedules. Hesitation to delay antibiotics until research samples are collected.	Perform virtual eConsenting when possible. Maximize virtual research encounters. Offer evening hours. Consider home nursing visits to document the examination, obtain vital signs and collect samples.
Hesitation to undergo any procedures (e.g. lumbar punctures, skin biopsies, additional blood draws) outside of what is necessary for routine clinical care.	Limit procedures when possible. Involve child life experts in study design and implementation. Carefully select tools and techniques that minimize discomfort during and after procedures.
Limited ability to quantify self-reported symptoms (e.g. fatigue, brain fog) in young individuals with limited language capabilities.	Utilize validated assessment tools specific to the age-specific population being studied. These exist for both self-reported and parent/guardian-reported symptoms.
Difficulty tolerating oral medications and formulations.	Ensure access to paediatric-friendly formulations including liquid solutions, dispersible powders, gummy bears and/or flavoured medications.
Need for child participant assent.	Discuss assent policy with local IRB/regulator. Consider how best to explain the child’s involvement in the study based on their age and developmental stage. Practice discussing the study with different age populations in advance.
Unplanned, child-specific concerns.	Include a child or child advocate on the study advisory board.
Difficulty recruiting child participants.	Establish research partnerships with local paediatricians, urgent care centres and emergency departments.

IRB = Institutional Review Board.

### Acquisition, transport and storage of samples related to Lyme disease and other IACIs

Collection, processing, storage and evaluation of biospecimens is a critical but often unrecognized stage in many clinical studies. Failure to properly obtain, transport, handle and store samples can lead to downstream errors with serious consequences. False-negative results can prevent assay development and deprive the community of a clinically valuable tool. False-positive results can lead to wrongful treatment of individuals without the disease, and false-negative results can prevent treatment of true patients.^173^ Whether collecting samples for research grants or using samples from a biorepository, the specific purpose needs to be defined and methods validated for trustworthy, reproducible results.^174^ The cost of irreproducible results is estimated at more than $US 28 billion/year.^175^ Irreproducibility incurs potential financial and outcome consequences. For example, in forensics, erroneous results can lead to the conviction of an innocent person and the evasion of justice by a guilty person.^176-180^

One entity that can aid a study in planning and implementing biospecimen collections and storage is an institutional biobank. Biobanks (or ‘biorepositories’) are valuable research partners that collect, process, store and distribute biospecimens.^181^ A biobank programme is the physical location and associated activities for operations, databases, consenting and regulatory documentation. Biobanks should provide high-quality, ethically collected, annotated specimens and associated data required to produce accurate, reproducible results. IACI researchers can collaborate with biobanks to help guide uniformity in specimen collection, processing and storage. Studies are encouraged to reference biobanking and collection planning considerations when implementing a study involving biospecimens for research analyses (Table 4). The overall goal in collection is to maintain high-quality biospecimens and decrease variability through well-designed methods and documentation.

**Table 4:** Considerations for planning a project that requires the collection of biospecimens for analysis and the associated ISBER best practices

Questions to cover in study design	Considerations^182^
What are the goals of the project?	This allows for the determination of end points, the required project length to meet those end points, and which specimens should be collected.
Who are the stakeholders of the project?	The stakeholders include the research team, collaborators, organizations, funders and regulators, etc. It may be necessary to create a collaborative agreement that details handoffs during the project lifecycle, roles and responsibilities, governance structures, funding sources, deposition of intellectual property and planned manuscripts. (BP A2.3)^182(p15)^
What type and numbers of biospecimens will be necessary for the intended assays?	The type and number will help plan for processing, storage and distribution. The number will be determined by statistical power calculations. It will also help determine barcode/schema, tube types and number of freezers needed for sample collection and storage. (BP A3.1.1)^182(p17)^
Which lab analyses will be needed to achieve the project’s goals?	Allows the determination of aliquot sizes for the processed specimens to minimize freeze-thaw cycles and maintain quality. Understanding the data being generated will help scale the data storage and analytical tools needed. (BP J3.8, I55; 12.2)^182(pp130,155)^
What quality standards must the specimens meet for valid laboratory assays?	It will help determine the pilot studies to be conducted and the quality control/quality assessment programme to be instituted. (BP D3.3)^182(p70)^
Have specimen collections processing and storage protocols been standardized and validated in pilot studies?	This includes packing, shipping, choice of transportation carrier(s), processing, aliquoting, labelling, storage, retrieval and emergency recovery. All pilot activities will support the buildout of robust, replicable processes for conducting your project. (BP J3.5)^182(p152)^
Where will the collection and processing sites be located?	Shipping protocols should be validated to ensure the stability and safety of specimens. Protocols should be shared and standardized across sites, with periodic training provided. (BP L3.2, L5.1)^182(pp177,180)^
How long will specimens be stored?	If specimens are stored for long periods, the stability of the intended biomarkers or analytes must be determined. This will include determining the required storage temperature. (BP J3)^182(p147)^
Do you have a plan for the logistics of labelling and coding of specimens?	The plan and logistics of labelling and coding of specimens and storage vessels must be determined up front. Schemas for labels or for pre-labelled tubes should be determined. This will allow for clean data linkage, ensure privacy for participants, and support operational movement of samples. (BP J3.1.4)^182(p149)^
Do you have a plan for the sustainability of your biobank?	Sustainability considerations, including financial, operational, and social aspects, are important to achieving the project’s goals. A plan to secure funding from start-up through the end points of the biobank is key. A plan should be created to allow for final disposition and sharing of the project data. (BP A3.2.2.3, A3.2.3)^182(pp21,22)^

ISBER = International Society for Biological and Environmental Repositories.

A key biobank best practice for mitigating introduced variability is the biospecimen ‘fit-for-purpose’ concept. The ‘fit-for-purpose’ concept indicates that the research purpose of the biospecimens and data is defined prior to collection, ensuring that the methods are suitable for the intended purposes.^154,183,184^ For example, shipping human blood specimens on ice packs can be suitable, with the specimens processed within 24 h of collection and stored long-term at appropriate temperatures (−80°C for blood fractions and −196°C for cells).^182,184^ These storage temperatures protect the biospecimens prior to -omics and cell-based assays by end users. An example of fit-for-purpose in Lyme disease studies is the CDC serologic biobank for the development of Lyme disease diagnostic tests. They required that serum samples be frozen at −20°C for no more than 2 weeks and thereafter maintained and shipped at −80°C until use.^185^ The CDC team determines that these temperatures are fit-for-purpose for developing antibody tests and state that the samples are not to be used for other purposes. IACI studies can implement the ‘fit-for-purpose’ concept to increase biospecimen research integrity.

Biobank standards and best practices can reduce sample variability introduced by discrepancies in sample management. The ISO/IEC General Requirements^186,187^ and ISBER Best Practices, 5th edition^182^ are relevant to any clinical study, as they guide uniformity in sample handling. A best practice when using biospecimens from a biobank or colleagues is to ask for the documentation on how the samples were collected, transported, processed and stored. Table 5 presents a partial list of sample handling dos and don’ts. These resources provide a framework for developing a uniform sample-handling plan for clinical studies. By following biobank best practices, IACI clinical trials ensure consistent biospecimen quality, enabling downstream use in pooled studies and meta-analyses.

**Table 5:** Sample collection dos and don’ts

Dos	Don’ts
Prior to collection, assess the feasibility of specimen availability, storage needs of specimens and limitations of storage capacity	Assume another group is able to store specimens for this project
Process specimens as rapidly as possible after collection, following preservation protocols, and according to the appropriate storage temperature for specific specimen types	Store samples in ‘temporary’ refrigerators if the goal is long-term preservation. Subject specimens to freeze-thaw-refreeze-rethaw cycles. Only thaw specimen portions that will be used
When retrieving and packaging specimens, maintain storage temperature throughout the process. Ensure compliance with and training in governing regulations for packaging and shipping hazardous materials (IATA, US Federal, etc.).	Ship and/or receive specimens without performing quality control checks to confirm all specimens are present in the package and in the stated conditions

IATA = International Air Transport Association.

### Statistical considerations

Scientific research, including clinical trials, epidemiological investigations, observational studies and case-control or cohort studies, involves three core steps: design, data analysis and results interpretation. Each is integrally related to the study’s key goals, outcomes, and hypotheses. Including a statistician at the beginning of project development is vital to the robustness of the study and to safeguarding consistency in results and, thus, the meaningfulness of conclusions. An expert in clinical statistical analysis provides the know-how to achieve this goal.

Study design involves calculation of the sample size needed to generate statistically meaningful results, proper experimental design and methods to collect the data, and the use of randomization and variables of control and other mechanisms to secure the optimal and appropriate use of hypothesis tests.

Data analysis includes the selection of necessary mathematical models, statistical methods and hypothesis tests to analyse the data as widely and deeply as possible, while avoiding preconceptions, minimizing missing data biases, pondering correlation structures and including random effects, among other complications. Modern techniques and methodologies, including machine learning and artificial intelligence-driven methods, are part of this process.

To interpret results and better display outcomes, a statistician’s intervention is crucial, as statistical significance and confidence intervals should accompany every finding and meaningful insight. Not including an expert statistician on the team could lead to a lack of accuracy, misinterpretation of the results and dismissal of the study’s findings by the scientific and medical community.

## Overall conclusions

We applied a series of questions to each of the above sections with emphasis on the ‘Dos’ and ‘Don’ts’. The major points are summarized in Table 6. By ‘Dos’, we refer to the necessary questions that must be addressed and the appropriate methods for doing so in a rigorous, reproducible and interpretable manner. By ‘Don’ts’, we highlight approaches that may be unsafe, unlikely to generate reproducible data, or prone to generating data that cannot be meaningfully interpreted within the broader context of the field beyond an individual study.

**Table 6:** Summary of highlights

	Dos	Don’ts
Study participant enrolment. Define entry criteria.	Have a clear, distinct and uniform method for identifying your population of interest that can be applied concurrently across all sites. Use objective, measurable criteria. Evaluate different phenotypes as separate groups. Consult a statistician early to ensure the sample size is sufficient to yield statistically meaningful data. Be realistic about enrolment numbers and ensure there are enough sites and personnel to achieve the goals. Consider the burden of participation on subjects and provide flexibility for study participants. Consider the samples to be collected and the feasibility of sample collection and handling. Consider fit-for-purpose. Establish stringent cut-offs for exclusion. Consult the IRB early in the process to ensure proper confidentiality and patient safety. If the process involves a potential FDA submission, be sure to consult the FDA about the suitability of the study prior to establishing it.	Enrol on the basis of subjective criteria, particularly if multiple sites. Use complex criteria that are difficult to apply to subjects. Mix different patient groups for analysis to reach a specific *n*. Set unreasonable expectations of patient enrolment numbers and excessive enrolment burden as this will drive down participation. Take all comers without first establishing grouping characteristics for analysis and conducting sample size calculations. Omit contingency plans for enrolment.
Control group considerations	Consider the relevance of controls. Statisticians and disease experts should be employed to ensure the quality and relevance of control populations. Consider matching variables beyond age and sex, such as BMI, living conditions/locality, working conditions and comorbidities (such as depression, anxiety, prior illnesses). For sample collection, consider complex variables, including time from infection (if known), time from onset of symptoms, the marker or metabolite being considered and the influence of outside factors such as sleep cycle and collection time.	Simply use a group of healthy volunteers, without considering the need for other controls to properly interpret data. Ignore the biology of the factors/metabolites/mediators being evaluated. Forget to consider the placebo effect. Enrol controls at a different time frame from the experimental groups.
Funding	Make sure the funding agency can provide funds for completion and publication.	Proceed without reasonable assurances that funds are there or encumbered for the study.

BMI = Body Mass Index; FDA = US Food and Drug Administration; IRB = Institutional Review Board.

Other considerations include ensuring there are sufficient resources, experienced investigators, patients, controls, and their samples, and adequate infrastructure. Factors that make a study unfeasible or impractical include insufficient numbers of patients with a clearly identifiable condition and inadequate financial support to perform the necessary work. Studies that are not safe are included in this category.

PTLDS and other IACIs are in desperate need of rigorously structured and executed clinical studies, with results supported by robust statistical analysis and interpreted in an unbiased way. In the case of PTLDS, it is apparent that studies that prospectively enrol participants prior to treatment, where at least one analysis group is restricted to those with direct microbiological proof of infection (i.e. where the presence of *B. burgdorferi* is unquestionable), are needed to establish causality and pathophysiology of resultant PTLDS. Other study participant groups can and likely should be included, but should be analysed independently of the microbiologically proven population. Patients suffering from PTLDS and CLD (those not meeting the definition of PTLDS) or other IACIs may not always ‘fit neatly into a clinical definition box’ but are nevertheless desperate to see and benefit from advances in treatment. In these cases, it is essential to avoid a heterogeneous co-mingling of samples of patients and instead include distinct groups within a study that can be stratified accordingly (e.g. confirmed versus suspected infection). Other important elements include the collection of metadata about the study participants. Where samples from study participants will be used for pathogenetic and diagnostic studies, they need to be obtained, transported and maintained in conditions that are fit for their purpose.

We recognize that many past studies were not as rigorously conducted as they could or should have been, since the more recent development of methods that can microbiologically prove that the initial infection occurred. Thus, the interpretation of the results may be called into question. Their data may still provide some investigative clues and insights upon which future prospective studies that incorporate definitive proof methodology can be based.

It is admirable that various scientific, medical and patient groups have recognized the added value of studying IACI commonalities so that each group can learn from the other. Not employing sound methods, such as microbiologic proof when the cause is known, or other methods that lend credibility to a study, would be a disservice to each disease and the people afflicted by them.

This paper outlines many key elements that will support clinical trials, ensuring results are interpretable and credible. Lyme disease and other affected communities will certainly have ideas that enhance our thoughts, and we hope to hear from them. We consider this an iterative process that can be improved as new data emerge.
